# Clinical Outcomes for Acute Kidney Injury in Acute Myocardial Infarction Patients after Intra-Aortic Balloon Pump Implantation: A Single-Center Observational Study

**DOI:** 10.31083/j.rcm2406172

**Published:** 2023-06-12

**Authors:** Xin-Ying Zhang, Zhong-Guo Fan, Hai-Mei Xu, Ke Xu, Nai-Liang Tian

**Affiliations:** ^1^Department of Cardiology, Nanjing First Hospital, Nanjing Medical University, 210006 Nanjing, Jiangsu, China; ^2^Department of Cardiology, Zhongda Hospital, School of Medicine, Southeast University, 210006 Nanjing, Jiangsu, China

**Keywords:** acute kidney injury, intra-aortic balloon pump, acute myocardial infarction, cardiogenic shock

## Abstract

**Background:**

Acute kidney injury (AKI) is common after cardiac interventional procedures. The prevalence and clinical outcome of AKI in patients with acute myocardial infarction (AMI) after undergoing intra-aortic balloon pump (IABP) implantation remains unknown. The aim of this study was to investigate the incidence, risk factors, and prognosis of AKI in specific patient populations.

**Methods:**

We retrospectively reviewed 319 patients with AMI between January 2017 and December 2021 and who had successfully received IABP implantation. The diagnostic and staging criteria used for AKI were based on guidelines from “Kidney Disease Improving Global Outcomes”. The composite endpoint included all-cause mortality, recurrent myocardial infarction, rehospitalization for heart failure, and target vessel revascularization.

**Results:**

A total of 139 patients (43.6%) developed AKI after receiving IABP implantation. These patients showed a higher incidence of major adverse cardiovascular events (hazard ratio [HR]: 1.55, 95% confidence interval [CI]: 1.06–2.26, *p* = 0.022) and an increased risk of all-cause mortality (HR: 1.62, 95% CI: 1.07–2.44, *p* = 0.019). Multivariable regression models found that antibiotic use (odds ratio [OR]: 2.07, 95% CI: 1.14–3.74, *p* = 0.016), duration of IABP use (OR: 1.24, 95% CI: 1.11–1.39, *p* < 0.001) and initial serum creatinine (SCr) (OR: 1.01, 95% CI: 1.0–1.01, *p* = 0.01) were independent risk factors for AKI, whereas emergency percutaneous coronary intervention was a protective factor (OR: 0.35, 95% CI: 0.18–0.69, *p* = 0.003).

**Conclusions:**

AMI patients who received IABP implantation are at high risk of AKI. Close monitoring of these patients is critical, including the assessment of renal function before and after IABP implantation. Additional preventive measures are needed to reduce the risk of AKI in these patients.

## 1. Introduction

Acute kidney injury (AKI) is a common occurrence after cardiac interventions, particularly in patients with baseline renal dysfunction, and results from improper or excessive use of contrast during the interventional procedure [1, 2]. A previous real-world study reported an AKI incidence of 11.6% and in-hospital mortality of 8.8% [3]. Several large cohort studies have also reported a higher incidence of AKI in patients with acute myocardial infarction (AMI) [4, 5, 6], suggesting that impaired renal function may be associated with worse clinical outcomes [7, 8, 9]. Moreover, the incidence of AKI increased to approximately 33% when complicated by cardiogenic shock (CS) [10], primarily due to the significant reduction in cardiac output [11, 12]. Current guidelines discourage intra-aortic balloon pump (IABP) implantation due to limited improvement in prognosis [13, 14]. IABP implantation is nevertheless thought to be helpful for stabilizing hemodynamics in some patients, and is commonly used in developing countries [15, 16]. Since peripheral blood flow in CS patients is further reduced by IABP implantation, this leads to further compromise of kidney function [17]. Therefore, additional studies are needed to determine the risk factors and clinical outcomes for AKI in AMI patients. To address this, we evaluated the incidence and risk factors for AKI in AMI patients who underwent IABP implantation.

## 2. Materials and Methods

### 2.1 Study Population

This was a single-center observational study of patients hospitalized at the coronary care unit, Nan Jing First Hospital, from January 2017 to December 2021. Patients were retrospectively assessed for eligibility using the following inclusion criteria: aged 18–85 years; diagnosis of AMI with CS; received successful IABP implantation. The diagnostic criteria for AMI and CS were based on prior descriptions [13, 18]. Patients with CS who were characterized by sustained hypotension (systolic blood pressure [SBP] <90 mmHg) in the presence of symptoms of hypoperfusion and appropriate filling status were recommended to receive IABP implantation. CS was deemed to be present if intravenous inotropes and/or mechanical support were required to keep the SBP >90 mmHg. The exclusion criteria were as follows: discharge with a diagnosis of unstable angina; death within 72 hours of admission; mechanical complications requiring transfer for additional surgery; diagnosis of malignant tumor; pregnancy; or severe liver dysfunction (serum aspartate aminotransferase or alanine aminotransferase >140 U/L). Electronic medical systems were used to extract information on baseline characteristics (demographics, vital signs, adjunctive medications, procedural details, laboratory and echocardiographic results) and clinical follow-up events.

### 2.2 Efficacy Endpoints and Relevant Definitions

Clinical follow-up was conducted through visits to the clinic or by telephone calls, ranging from 1 to 18 months after discharge. The composite endpoint was a major adverse cardiovascular event (MACE), which included all-cause mortality, recurrent myocardial infarction, rehospitalization for heart failure, and target vessel revascularization. Based on guidelines from the Kidney Disease Improving Global Outcomes (KDIGO) [19], the diagnostic criteria for AKI used in the present study was an increase in the serum creatinine (SCr) concentration by ≥0.3 mg/dL within 48 h, or an increase in the SCr concentration to ≥1.5 times that of baseline within 7 days. Different stages of AKI were also distinguished according to the SCr concentration and urine output, and these were classified as stages 0 to 3 [19]. The estimated glomerular filtration rate (eGFR) was calculated using the method described by the Chronic Kidney Disease-Epidemiology Collaboration (CKD-EPI) [20].

### 2.3 Procedures and Medications

Experienced interventionists oversaw all procedures following accepted standards. The use of glycoprotein IIb/IIIa inhibitors, pre-dilation or post-dilation, and the type of implanted drug eluting stent (DES) were at the discretion of the interventionist. A loading dose of clopidogrel (300 mg) or ticagrelor (180 mg) was routinely administered prior to percutaneous coronary intervention (PCI) procedures. A standard dual antiplatelet therapy consisting of aspirin (100 mg/d) and a ${\mathrm{P2Y}}_{12}$ inhibitor [clopidogrel (90 mg daily) or ticagrelor (90 mg bid)] was recommended for at least 12 months post-PCI. A successful PCI procedure was defined as a thrombolysis in myocardial infarction grade 3 and residual stenosis of <10%. Conventional doses only of contrast agent were used for each patient during the PCI procedure, and all patients received hydration (0.5–1 mL/kg/h) before and after PCI for at least 24 hours. Unfractionated heparin was used for perioperative anticoagulation and X-ray was performed daily to ensure the correct position of the implanted balloon. The IABP should be removed immediately as soon as any IABP-related complications occur. Emergency PCI was defined as primary PCI performed within 12 h of the onset of myocardial infarction. Statins, β-blockers, aldosterone antagonists, angiotensin-converting enzyme inhibitors or sodium–glucose co-transporter 2 inhibitors were commonly recommended as adjunctive therapies for secondary prevention according to the current guidelines. SCr concentrations were monitored until the patient was discharged.

### 2.4 Statistical Analysis

Continuous variables were expressed as the mean ± standard deviation, or as the median with inter-quartile range as appropriate. Differences in normally distributed data were compared using the student’s *t*-test. The Mann-Whitney U test was used for analysis of data that was not normally distributed. Categorical variables were displayed as counts with percentages, and the Fisher’s exact test or ${\mathrm{chi}}^{2}$ test was used to evaluate differences between the two groups. All *p*-values were two-tailed and a *p*-value < 0.05 was considered to be statistically significant. Binary logistic regression analysis was performed to exclude confounding factors and to identify independent predictors for AKI. Kaplan-Meier survival curves were generated for all-cause mortality and MACEs, with the log-rank test used for comparisons. All data were analyzed using SPSS software (version 22.0, SPSS Institute, Chicago, IL, USA) or GraphPad Prism 8 (GraphPad Software, La Jolla, CA, USA).

## 3. Results

### 3.1 Baseline and Clinical Characteristics of the Study Population

**Fig. 1.**
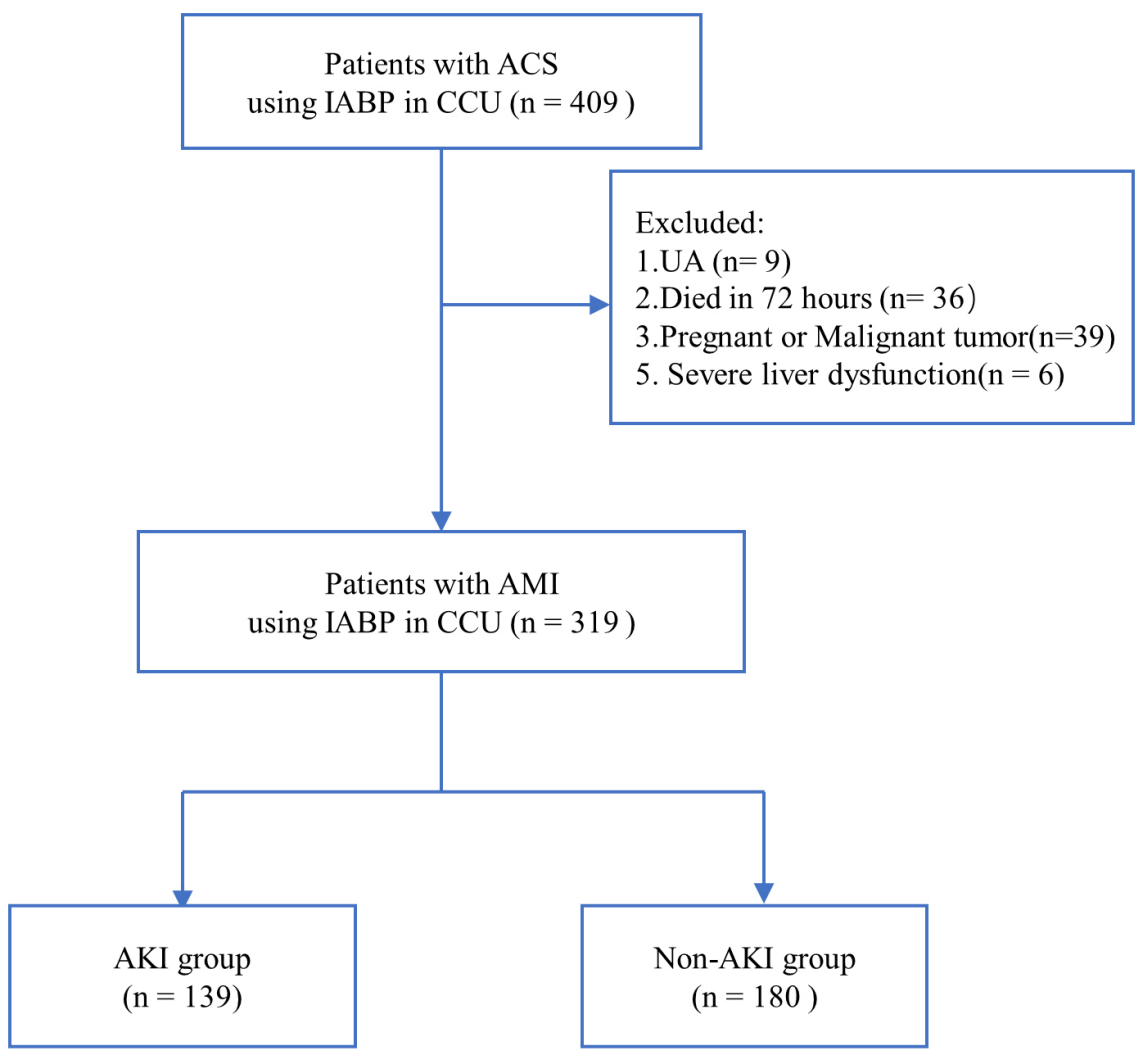
**Flow chart showing patient selection**. ACS, acute coronary syndrome; CCU, coronary care unit; UA, unstable angina; AMI, acute myocardial infarction; IABP, intra-aortic balloon pump; AKI, acute kidney injury.

**Fig. 2.**
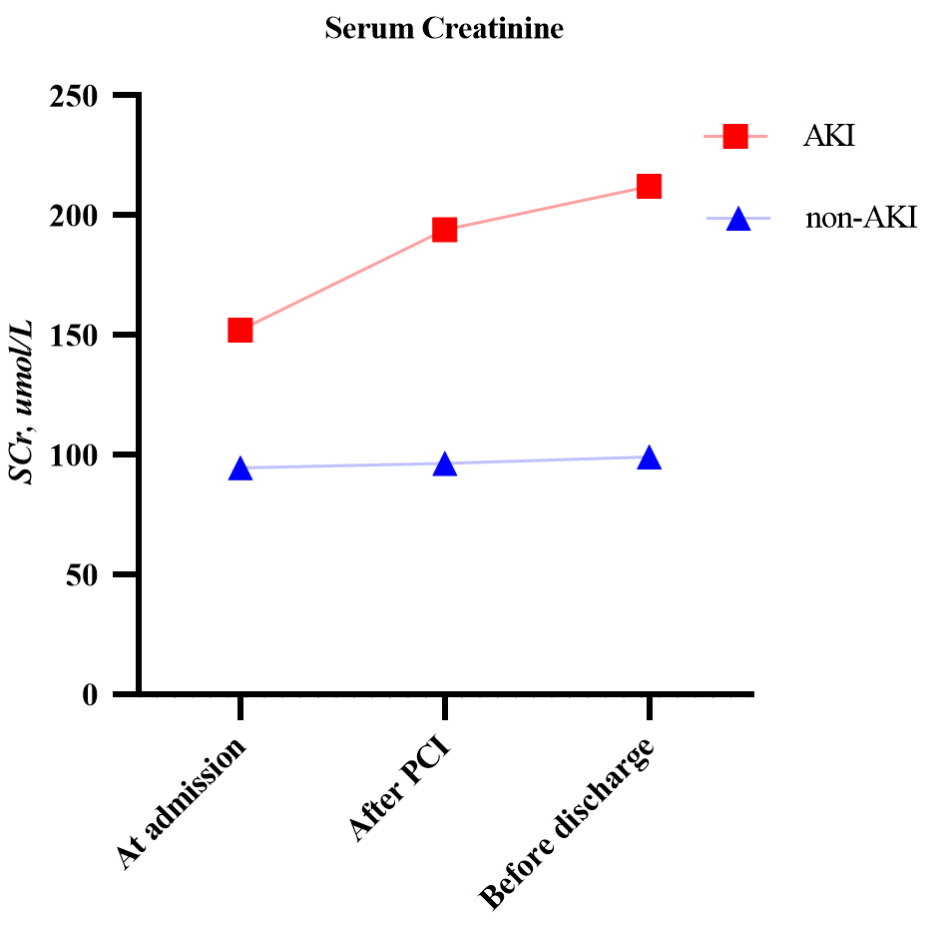
**Changes in serum creatinine in the AKI and non-AKI groups**. AKI, acute kidney injury; PCI, percutaneous coronary intervention.

**Fig. 3.**
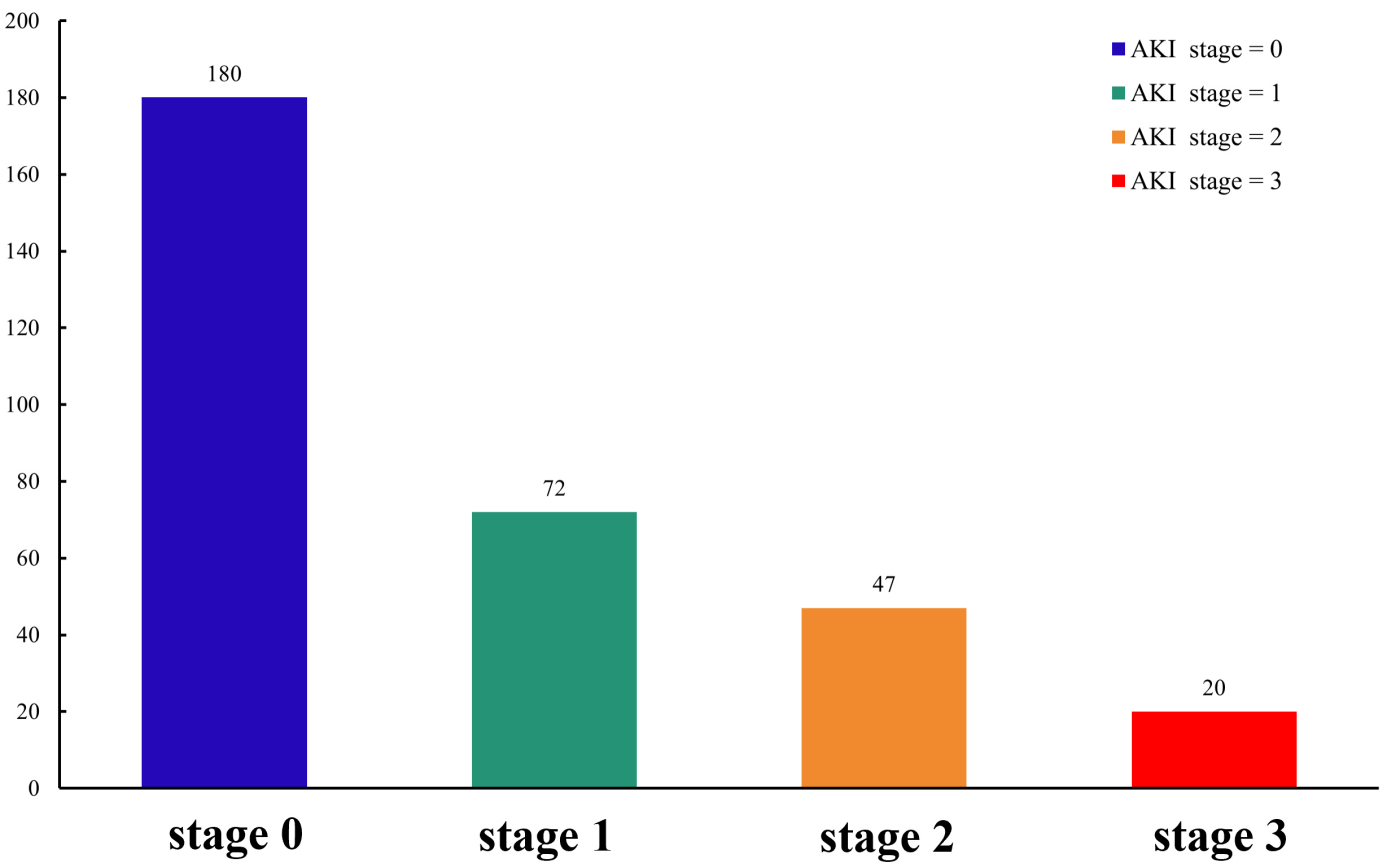
**Stage of AKI in the study population**. AKI, acute kidney injury

A total of 319 consecutive AMI patients who underwent successful IABP implantation were included in the study cohort. Of these, 139 (43.6%) were diagnosed with AKI after receiving IABP implantation, while remaining patients were classified as the non-AKI group (n = 180). The flow chart used for the selection of study participants is shown in Fig. 1, while the baseline characteristics of eligible patients are summarized in Table 1. Significant differences in heart rate (HR) and Killip classifications were observed between the AKI and non-AKI groups. Additionally, patients with AKI had a significantly increased incidence of ventricular fibrillation and were more likely to be treated with vasopressors (58.3% vs. 40.6%, *p =* 0.002) and antibiotics (69.1% vs. 39.4%, *p* < 0.001). The SCr, blood urea nitrogen, serum potassium and serum phosphorus levels were also significantly higher in the AKI group, whereas the eGFR and serum albumin levels were lower than in the non-AKI group. The changes in SCr levels in both groups are shown in Fig. 2. The majority of patients in the AKI group suffered mild-moderate renal injury (stages 1–2), although 14.4% were diagnosed with severe renal injury (stage 3, Fig. 3).

**Table 1.** **Baseline characteristics of the study population**.

Characteristic		Non-AKI (N = 180)	AKI (N = 139)	*p* value
Age, yrs		70.0 (61.0 to 77.0)	69.0 (60.5 to 77.0)	0.735
Male, n%		135 (75%)	108 (78.3%)	0.585
Hypertension, n%		132 (73.3%)	91 (65.5%)	0.163
Diabetes		71 (39.4%)	63 (45.3%)	0.347
Stroke, n%		30 (16.8%)	31 (22.3%)	0.271
Dyslipidemia, n%		110 (61.1%)	84 (60.4%)	0.994
BMI, kg/$m^{2}$		23.7 (21.6 to 25.7)	23.9 (21.4 to 26.0)	0.610
HR, bpm		88.3 ± 21.9	93.3 ± 21.3	0.041
SBP, mmHg		122.3 ± 25.0	120.5 ± 23.3	0.512
DBP, mmHg		78.0 (68.0 to 89.0)	77.0 (68.0 to 90.0)	0.898
Killip classification				0.015
	Killip I	54 (30.3%)	28 (20.1%)	
	Killip II	50 (28.1%)	31 (22.3%)	
	Killip III	17 (9.6%)	27 (19.4%)	
	Killip IV	57 (32%)	53 (38.1%)	
LVEF, %		45.0 (37.0 to 52.0)	44.0 (34.0 to 50.0)	0.106
STEMI, n%		120 (66.7%)	94 (67.6%)	0.952
CRRT, n%		4 (2.2%)	2 (1.4%)	0.700
Ventricular fibrillation, n%		23 (12.8%)	34 (24.5%)	0.011
Antibiotic administration, n%		71 (39.4%)	96 (69.1%)	<0.001
Vasopressor administration, n%		73 (40.6%)	81 (58.3%)	0.002
BUN, mmol/L		8.3 (5.7 to 12.4)	9.5 (7.1 to 14.6)	0.006
SCr, umol/L		81.2 (67.2 to 106.0)	117.0 (80.3 to 175.6)	<0.001
eGFR, mL/min		134.3 (98.4 to 155.4)	103.7 (61.3 to 135.6)	<0.001
Serum albumin, g/L		36.1 (33.8 to 38.7)	34.6 (32.1 to 37.3)	<0.001
Serum potassium, mmol/L		3.9 (3.7 to 4.2)	4.1 (3.8 to 4.4)	0.011
Serum phosphorus, mmol/L		1.1 (0.9 to 1.4)	1.2 (1.0 to 1.5)	<0.001

AKI, acute kidney injury; BMI, body mass index; HR, heart rate; SBP, systolic blood pressure; DBP, diastolic blood pressure; LVEF, left ventricular eject fraction; STEMI, ST segment elevated myocardial infarction; CRRT, continuous renal replacement therapy; BUN, blood urea nitrogen; SCr, serum creatinine; eGFR, estimated glomerular filtration rate.

### 3.2 Procedural Details for the Study Population

The procedural details for the two patient groups are shown in Table 2. Patients in the non-AKI group underwent PCI more often than the AKI group (86.7% vs. 77% respectively, *p =* 0.035), of which 39.1% received an emergency procedure. However, there was no difference in the number of implanted DES (2.0 vs. 2.0, *p =* 0.872) between the two groups. There were also no significant differences in the use of glycoprotein IIb/IIIa inhibitors or in the dose of infused contrast. The AKI group had a significantly longer duration of IABP (7 vs. 5 days, *p <* 0.001).

**Table 2.** **Procedural details for the AKI and non-AKI groups**.

Characteristic		Non-AKI (N = 180)	AKI (N = 139)	*p* value
CAG, n%		161 (89.4%)	110 (79.1%)	0.017
PCI, n%		156 (86.7%)	107 (77%)	0.035
Emergency PCI, n%		63 (39.1%)	21 (19.3%)	<0.001
Number of stents		2.0 (1.0 to 2.0)	2.0 (1.0 to 2.0)	0.872
Culprit artery, n%				0.358
	LM	26 (15.7%)	20 (16.4%)	
	LAD	83 (50.9%)	68 (55.8%)	
	LCX	13 (8.0%)	12 (9.8%)	
	RCA	41 (25.2%)	22 (18.0%)	
Number of lesion vessels				0.334
	1	28 (17.8%)	13 (12.1%)	
	2	42 (26.8%)	26 (24.3%)	
	3	87 (55.4%)	68 (63.6%)	
CTO, n%		41 (26.1%)	31 (28.4%)	0.780
Contrast volume, mL		160.0 (140.0 to 200.0)	150.0 (130.0 to 220.0)	0.824
Heparin, IU		7000 (5775, 8500)	7230 (6000, 9050)	0.248
Bivalirudin, n%		75 (49%)	44 (40.7%)	0.232
Tirofiban, n%		46 (29.3%)	22 (20.2%)	0.125
Duration of PCI, min		40.0 (30.0 to 60.0)	40.0 (25.0 to 65.0)	0.984
Duration of IABP use, days		5.0 (4.0 to 6.0)	7.0 (5.0 to 11.0)	<0.001

AKI, acute kidney injury; CAG, coronary angiography; PCI, percutaneous coronary intervention; LM, left main coronary artery; LAD, left anterior descending coronary artery; LCX, left circumflex artery; RCA, right coronary artery; CTO, chronic total occlusion; IABP, intra-aortic balloon pump.

### 3.3 Clinical Outcomes

**Fig. 4.**
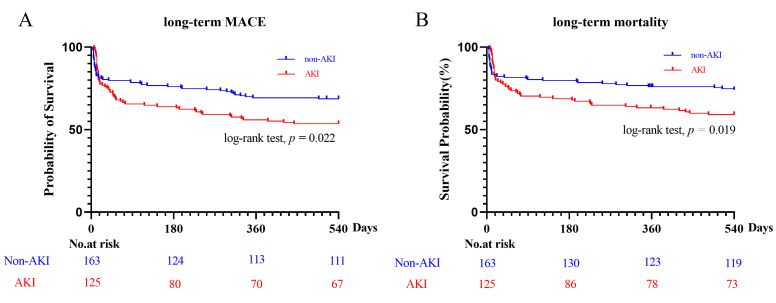
**Kaplan-Meier analysis of MACE (A) and mortality (B) in non-AKI and AKI groups**. MACE, major adverse cardiovascular event; AKI, acute kidney injury.

**Fig. 5.**
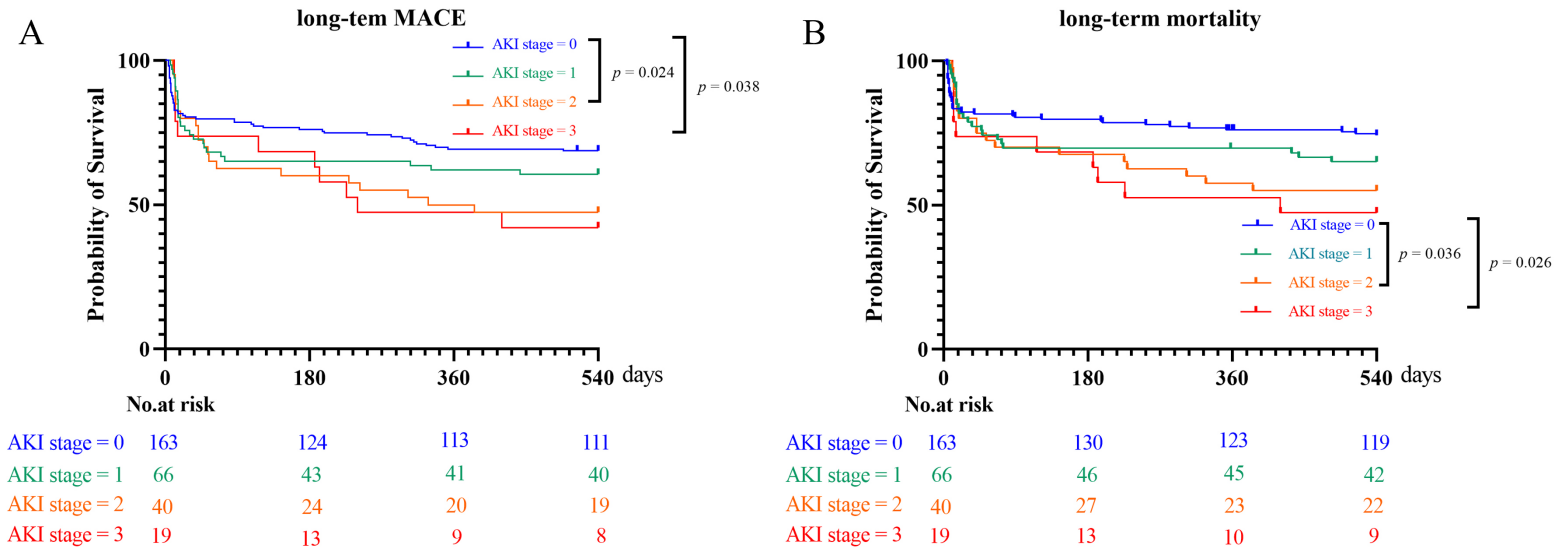
**Kaplan-Meier analysis of MACE (A) and mortality (B) in patients with different AKI stages**. MACE, major adverse cardiovascular event; AKI, acute kidney injury.

After an 18-month follow-up period, no significant differences were observed between the two groups for the incidence of recurrent myocardial infarction (AKI vs. non-AKI: 1.4% vs. 1.7%, *p =* 0.871), rehospitalization for heart failure (5.3% vs. 3.9%, *p =* 0.620) and target vessel revascularization (3.8% vs. 3.4%, *p =* 0.957) (Table 3). However, Kaplan–Meier analyses found that patients with AKI had a higher risk of MACEs than those without AKI (41.7% vs. 28.3%, *p =* 0.022, Fig. 4A), which likely also contributed to the higher incidence of all-cause mortality (36.7% vs. 23.3%, *p =* 0.019, Fig. 4B). Kaplan–Meier analyses were also performed according to the stage of AKI. The severity of AKI was found to be associated with an increased risk of MACEs (stage 2 vs. stage 0: *p* = 0.024; stage 3 vs. stage 0: *p* = 0.038, Fig. 5A), which contributed to an increased incidence of mortality (stage 2 vs. stage 0: *p* = 0.036; stage 3 vs. stage 0: *p* = 0.026, Fig. 5B).

**Table 3.** **Clinical outcomes in the AKI and non-AKI groups**.

Outcome	Total (N = 319)	non-AKI (N = 180)	AKI (N = 139)	HR (95% CI)	*p* value
MACE, n%	109 (34.2%)	51 (28.3%)	58 (41.7%)	1.55 (1.06–2.26)	0.022
All-cause Mortality, n%	93 (29.1%)	42 (23.3%)	51 (36.7%)	1.62 (1.07–2.44)	0.019
recurrent myocardial infarction, n%	5 (1.6%)	3 (1.7%)	2 (1.4%)	0.86 (0.14–5.23)	0.871
rehospitalization for heart failure, n%	14 (4.4%)	7 (3.9%)	7 (5.3%)	1.31 (0.45–3.83)	0.620
target vessel revascularization, n%	9 (2.8%)	5 (3.4%)	4 (3.8%)	1.04 (0.27–3.94)	0.957
CABG, n%	5 (1.6%)	4 (2.2%)	1 (0.7%)	0.32 (0.03–2.89)	0.319

AKI, acute kidney injury; HR, hazard ratio; CI, confidence interval; MACE, major adverse cardiovascular events; CABG, coronary artery bypass grafting.

### 3.4 Logistic Regression Analyses

As shown in Table 4, emergency PCI was protective against AKI (odds ratio [OR]: 0.35, 95% CI: 0.18–0.69, *p* = 0.003). In contrast, antibiotic administration (OR: 2.07, 95% CI: 1.14–3.74, *p =* 0.016), duration of IABP use (OR: 1.24, 95% CI: 1.11–1.39, *p* < 0.001) and initial SCr (OR: 1.01, 95% CI: 1.00–1.01, *p =* 0.01) were potential risk factors for AKI in the study population.

**Table 4.** **Logistic Regression Analyses of Risk Factors for AKI**.

Characteristic	Univariable		Multivariable	
	OR (95% CI)	*p* value	OR (95% CI)	*p* value
HR	1.01 (1–1.02)	0.042		
Killip I	Ref			
Killip II	1.46 (0.74–2.89)	0.276		
Killip III	3.48 (1.43–8.43)	0.006		
Killip IV	1.90 (0.97–3.69)	0.060		
Ventricular Fibrillation	2.34 (1.20–4.56)	0.012	1.91 (0.88–4.13)	0.101
Emergency PCI	0.35 (0.20–0.64)	<0.001	0.35 (0.18–0.69)	0.003
Vasopressor administration	2.05 (1.31–3.22)	0.002		
Antibiotic administration	3.62 (2.14–6.10)	<0.001	2.07 (1.14–3.74)	0.016
Duration of IABP use	1.28 (1.15–1.41)	<0.001	1.24 (1.11–1.39)	<0.001
BUN	1.05 (1.01–1.09)	0.016		
SCr	1.01 (1.01–1.02)	<0.001	1.01 (1.00–1.01)	0.010
Serum Albumin	0.93 (0.87–0.99)	0.027		
Serum Phosphorus	2.38 (1.15–4.92)	0.019		

AKI, acute kidney injury; OR, odds ratio; CI, confidence interval; HR, heart rate; VSR, ventricular septal rupture; PCI, percutaneous coronary intervention; IABP, intra-aortic balloon pump; BUN, blood urea nitrogen; SCr, serum creatinine.

## 4. Discussion

This retrospective study the assessed clinical outcomes of patients with AMI who underwent successful IABP implantation. The major finding was that the presence of AKI in these patients significantly increased the incidence of MACEs and all-cause mortality. Moreover, a higher severity of AKI was associated with worse prognosis, and the duration of IABP use was found to be an independent predictor of AKI.

It is well established that ischemia and the utilization of nephrotoxic drugs are the two main causes of AKI in the clinic [21, 22]. AMI patients may be more vulnerable to AKI because they have significantly decreased cardiac output and more hemodynamic disorders, especially to the extent seen in CS [10]. Currently, IABP is recommended for CS patients in order to stabilize their hemodynamics [15, 16], despite the possibility of peripheral perfusion deficiency. However, the improper or excessive use of contrast during PCI procedures can exacerbate kidney injury in such patients [23, 24]. Therefore, it is important to identify the potential risk factors for AKI in these specific populations so that their clinical outcome can be improved. The present study was conducted to assess the clinical outcome of AMI patients after receiving successful IABP implantation.

We found the incidence of AKI in this specific patient population was approximately 43.6%. This was likely to have contributed to the increased incidence of all-cause death in AKI patients compared to non-AKI patients (36.7% vs. 23.3% respectively, *p =* 0.019). An earlier study reported that AKI occurred in almost one third of patients during the first day of CS following AMI [10]. However, this study did not exclude patients who died within 72 hours after admission. Moreover, the proportion of patients who underwent coronary angiography (34.7%) or PCI (16.9%) was much lower than in our study (85.0% and 82.4%, respectively). It is important to note that the association between mechanical circulatory assist devices and renal injury remains controversial. IABP is a commonly used cardiac assist equipment for CS and can improve cardiac output to a certain degree, but with the potential risk of renal hypoperfusion [17]. Achieving a balance between the use of IABP and renal hypoperfusion remains challenging. Several earlier studies reported that mechanical circulatory support (MCS) was the preferred option for patients with CS complicated by kidney injury [25, 26]. Moreover, another study suggested that early implantation in conjunction with coronary circulation reconstruction led to improved prognosis in these patient subsets [27]. Nonetheless, longer utilization of MCS has also been associated with a higher incidence of AKI [28]. Our results also showed that long-term IABP use was an independent risk factor for AKI in these patients. In contrast, emergency PCI was associated with a lower incidence of AKI and was a protective factor in these patients. This may have been a result of improved cardiac function following revascularization. The use of different interventional strategies and patient cohorts with different baseline characteristics could be expected to influence the clinical outcomes. A total of 97 consecutive patients diagnosed with ST segment elevated myocardial infarction and complicated by CS were enrolled in an earlier cohort study [8]. The incidence of AKI amongst these patients was 55%, with binary logistic regression analysis also identifying the initial SCr level as an independent risk factor for AKI.

Several other studies have reported potential risk factors for AKI in AMI patients [29, 30, 31]. The SILVER-AMI study found that HR, left ventricle eject fraction, body mass index, creatinine clearance, presentation of heart failure, prior myocardial infarction and initial hemoglobin were independent risk factors for AKI [30]. An observational study found that hospital-acquired infection, NT-proBNP and prior resuscitation were significantly correlated with acute kidney damage [29]. Several other studies have suggested that age, hypertension, diabetes, chronic kidney disease phase, Killip classification, and extensive anterior myocardial infarction were potential risk factors for kidney impairment [31]. Additionally, the present study found that antibiotic use was an independent risk factor for AKI after adjusting for ventricular fibrillation.

In order to correctly implement the many preventive strategies for AKI, clinicians should take into account the individual characteristics of each patient. Currently, revascularization of the culprit vessel is still strongly recommended for these AMI patients [13, 32]. The risk of contrast-induced AKI in AMI could also be estimated using several clinical prediction models [23, 33]. According to the different risk stratifications, forced diuresis with matched hydration can help prevent AKI following PCI procedures in AMI patients [34, 35, 36]. PCI may therefore be beneficial for patients diagnosed with CS, since fluid management and homeostasis of the inner environment is more challenging in these patients due to the vulnerability of cardiac pump function. CS patients are also more likely to have co-morbidities such as diabetes, stroke and chronic kidney disease. Indeed, several earlier studies have suggested that diabetes can cause an over-inflammatory and thrombotic status [37, 38, 39]. This arises because of severe endothelial injury, which then significantly increases the risk of diabetic vascular complications and leads to poor prognosis, especially in AMI patients. The complete loss of blood flow to the myocardium results in a large amount of cardiomyocyte death, triggering significantly lower cardiac output and the development of hemodynamic disorders. The therapeutic tools available for such patient groups with diabetes are then limited [10, 40]. Treatment with sodium-glucose co-transporter 2 inhibitor (SGLT2i) has been reported to have marked cardioprotective benefits in such patients [38, 41, 42]. This new oral antidiabetic agent has been associated with many potential mechanisms of action. A widely accepted concept is that SGLT2i reduces myocardial apoptosis and the secretion of inflammatory cytokines after AMI. This could help to repair endothelial damage and stabilize hemodynamics, thereby preserving renal function and thus improving prognosis [43]. To prevent AKI, it could therefore be useful to mitigate various risk factors by monitoring serum glucose, lipid levels, signs of infection, and SCr levels.

## 5. Limitations

There are several limitations to our study. First, this was a single-center, retrospective study and hence some selection bias may have occurred. Larger, prospective randomized trials are warranted to overcome this. Second, although we applied the KDIGO criteria to diagnose AKI, several different diagnostic criteria exit and this can influence the selection of patients with AKI. Finally, the lack of information on the use of hypoglycemic medications limited our ability to explore the potential effects of diabetes on AKI.

## 6. Conclusions

The results of this study indicate that AKI is associated with a significantly increased risk of all-cause death in AMI patients after IABP implantation. Moreover, a higher severity of AKI is associated with poorer prognosis in these patients. The administration of antibiotics, the duration of IABP use, and the initial SCr level were identified as independent risk factors for AKI, whereas emergency PCI was found to be a protective factor. Finally, renal function should be assessed both before and after IABP implantation in AMI patients so as to facilitate the identification of patients who are at high-risk of AKI, thereby allowing intervention.

## Data Availability

All data generated or analyzed during this study are included in this published article.
